# Removal of Siloxanes from Model Biogas by Means of Deep Eutectic Solvents in Absorption Process

**DOI:** 10.3390/ma14020241

**Published:** 2021-01-06

**Authors:** Edyta Słupek, Patrycja Makoś-Chełstowska, Jacek Gębicki

**Affiliations:** Department of Process Engineering and Chemical Technology, Faculty of Chemistry, Gdansk University of Technology, G. Narutowicza St. 11/12, 80-233 Gdańsk, Poland; edyta.slupek@pg.edu.pl (E.S.); jacek.gebicki@pg.edu.pl (J.G.)

**Keywords:** absorption, biogas, deep eutectic solvents, siloxanes

## Abstract

The paper presents the screening of 20 deep eutectic solvents (DESs) composed of tetrapropylammonium bromide (TPABr) and glycols in various molar ratios, and 6 conventional solvents as absorbents for removal of siloxanes from model biogas stream. The screening was achieved using the conductor-like screening model for real solvents (COSMO-RS) based on the comparison of siloxane solubility in DESs. For the DES which was characterized by the highest solubility of siloxanes, studies of physicochemical properties, i.e., viscosity, density, and melting point, were performed. DES composed of tetrapropylammonium bromide (TPABr) and tetraethylene glycol (TEG) in a 1:3 molar ratio was used as an absorbent in experimental studies in which several parameters were optimized, i.e., the temperature, absorbent volume, and model biogas flow rate. The mechanism of siloxanes removal was evaluated by means of an experimental FT-IR analysis as well as by theoretical studies based on σ-profile and σ-potential. On the basis of the obtained results, it can be concluded that TPABr:TEG (1:3) is a very effective absorption solvent for the removal of siloxanes from model biogas, and the main driving force of the absorption process is the formation of the hydrogen bonds between DES and siloxanes.

## 1. Introduction

The production of energy from renewable sources is not only a choice resulting from the policy of environmental protection or care of the environment but is also an obligation imposed by the European Union in the form of numerous ordinances and international agreements [1]. Therefore, more and more EU countries are focusing their attention on managing waste materials from various industries for the production of biogas [2,3,4,5]. This approach is consistent with the theory of sustainable development. However, the obtained biogas is usually a multicomponent mixture containing both inorganic and organic substances, i.e., methane (30–60% *v/v*), carbon dioxide (15–30% *v/v*), water, ammonia, hydrogen sulfide, organosulfur compounds, siloxanes, and other linear and aromatic volatile organic compounds (VOCs) [6,7].

The chemical composition of the waste biogas changes depending on the type of raw materials used in the dark fermentation process. The presence of gaseous substances other than methane causes many technological and environmental problems. Particularly dangerous pollutants include siloxane compounds, which can appear in the biogas from municipal landfills or wastewater treatment plants [8,9]. During the combustion of such types of biogas, silicone may be released and combined with oxygen. This can lead to the formation of silica deposits. The silica deposits can cause abrasion of engine parts or the formation of layers that inhibit thermal conductivity or lubrication and clogged transmission lines [10]. Therefore, in order to eliminate the failure of engines converting biogas into energy and to meet the quality requirements for fuels, raw biogas must undergo several treatment processes. The oldest and most widely used process for the treatment of gaseous streams is the application of water or amine scrubbers [11,12]. However, most siloxanes are hydrophobic, and only some of them, i.e., trimethylsilanol, can be absorbed with water because of their high solubility therein [13,14]. Amine scrubbers do not show satisfactory efficiency of siloxane removal either. Among the effective absorbents, there are mineral oils, mixtures of glycols, or inorganic acids [15,16,17,18]. Although the abovementioned absorption methods allow for the recovery of solvents, these methods have a significant disadvantage, which is their energy consumption resulting from the large amount of energy needed to regenerate the absorbent. Therefore, in recent years, more and more scientific research has been devoted to the search for new “green solvents” that will have higher purification efficiency of biogas streams with a simultaneous lower energy demand during regeneration [19].

In the last few years, ionic liquids (ILs) have attracted a lot of attention because they belong to the class of new solvents with a high affinity for CO_2_ and a wide range of VOCs [20,21] In addition, ILs have a lower degradation rate, a lower energy requirement for solvent regeneration, and lower corrosive characteristics compared to conventional amine-based solvents [22]. The main disadvantages of ILs are their high viscosity, very high prices, and toxic character. Therefore, deep eutectic solvents (DESs) are a good alternative to ILs because they are much cheaper, less toxic, and more biodegradable [23]. These advantageous properties have made DESs widely used in various separation processes such as extraction [24,25,26,27], absorption [28,29,30,31,32,33], or adsorption [34]. So far, DES has not been used for the experimental removal of siloxanes from biogas. Only theoretical studies can be found in the literature [35].

The study presents screening of twenty-five deep eutectic solvents composed of tetrapropylammonium bromide (TPABr) as hydrogen bond acceptor (HBA) and glycols as hydrogen bond donors (HBDs) in various molar ratio as absorbents for removal of siloxanes from model biogas stream. For this proposal, the conductor-like screening model for real solvents (COSMO-RS) was used. The selection of DESs with the highest siloxane capacity potential was made on the basis of the calculated solubility. For DES (TPABr:TEG 1:3), which was characterized by the highest solubility of siloxanes, the study of its physicochemical properties, i.e., viscosity, density, and the melting point, was performed. Further on, optimization studies of the main parameters influencing the absorption processes were carried out. The mechanism of siloxane removal was evaluated by means of an experimental FT-IR analysis as well as theoretical studies based on σ-profile and σ-potential. To the best of our knowledge, this is the first study dedicated to the application of DES for experimental removal of siloxanes from the gas steams.

## 2. Materials and Methods

### 2.1. Materials

The following pure substances were used in this study: tetrapropylammonium bromide (TPABr) (purity ≥ 99.0%), tetraethylene glycol (TEG) (purity 99%), hexamethyldisiloxane (L2) (purity 98.5%), octamethyltrisiloxane (L3) (purity 98.5%), and octametylocyclotetrasiloxane (D4) (purity 98%) were purchased from Sigma Aldrich (St. Louis, MO, USA).

For the preparation of model biogas, compressed gases such as nitrogen (purity *N* 5.5) and methane (purity *N* 5.0) (Linde Gas, Łódź, Poland) were used. Additionally, for the GC analysis, compressed gases such as nitrogen (purity *N* 5.5), air (purity *N* 5.0) generated by a DK50 compressor with a membrane dryer (Ekkom, Cracow, Poland), and hydrogen (purity *N* 5.5) generated by a 9400 Hydrogen Generator (Packard, Detroit, MI, USA) were used.

### 2.2. Apparatus

The purification process was controlled by gas chromatography (Autosystem XL) (PerkinElmer, Waltham, MA, USA) coupled with a flame ionization detector (FID) (PerkinElmer, Waltham, MA, USA) and an HP-5 (30 m × 0.25 mm × 0.25 μm) capillary column (Agilent Technologies, Santa Clara, CA, USA). In the investigations, the TurboChrom 6.1 software (PerkinElmer, Waltham, MA, USA), was used.

The following apparatus was used to evaluate the physicochemical properties: Bruker Tensor 27 spectrometer (Bruker, Billerica, MA, USA) with an ATR accessory and OPUS software (Bruker); BROOKFIELD LVDV-II  +  viscometer (Labo-Plus, Warsaw, Poland); DMA 4500 M (Anton Paar, Graz, Austria).

### 2.3. Procedures

#### 2.3.1. COSMO-RS Studies

The geometry optimization of TPABr:TEG (1:3) was performed by means of the continuum solvation COSMO model at the BVP86/TZVP level of theory. The level of theory was used based on previous studies [35,36]. Multiple starting geometries of TPABr:TEG (1:3) were created and optimized in the gas phase to identify stable conformers. In the next step, the vibrational analysis was conducted to find the DES conformer correspond to the true energy minimum. Full geometry optimization was performed only for the most energetically favorable conformer.

In the studies, the COSMO-RS model was used for the screening of DESs using ADF COSMO-RS software (SCM, Netherlands). The relative solubility of siloxanes (xj) in DESs were calculated using Equation (1):

(1) $$log_{10}\left(x_{j}\right)=log_{10}\left[\frac{\exp\left(\mu_{j}^{pure}-\mu_{j}^{solvent}-∆G_{j,fusion}\right)}{RT}\right]$$

where: $\mu_{j}^{pure}$—chemical potential of pure siloxanes (J/mol); $\mu_{j}^{solvent}$—chemical potential of siloxanes at infinite dilution (J/mol); $∆G_{j,fusion}$—fusion free energy of siloxanes (J/mol); R—universal gas constant = 8.314 (J/mol·K); T—temperature (K) [37,38,39].

#### 2.3.2. Preparation of DES

The deep eutectic solvent was successfully synthesized by mixing TPABr and TEG in 1:3 molar ratio, on a magnetic stirrer under 800 rpm, at 80 °C. All components were dried in a vacuum oven before mixing. The mixing process was carried out for half an hour. The resulting liquid DES was left cooling to room temperature (RT).

#### 2.3.3. Preparation of Model Impurities and Biogas

The model impurities were prepared by means of the barbotage process. Pure nitrogen was moved through a vial containing 1 mL of each siloxane. The obtained model impurities were diluted with a nitrogen stream to acquire a suitable concentration of siloxanes (50 mg/dm^3^). This is the upper limit of the range of siloxane concentrations which can be identified in biogas [40].

The model biogas stream was prepared in two options. The first with the use of pure nitrogen, and the second with the use of a mixture of nitrogen and methane gases in the volume ratio of 2:1.

#### 2.3.4. Absorption Process

**Figure 1:**
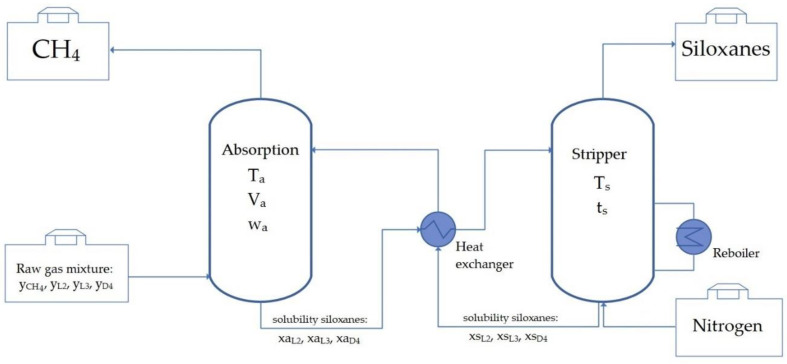
Simplified process flow diagram of siloxanes separation using deep eutectic solvent (DES).

The installation to separate the siloxanes consists of an absorption column, a stripper column, a heat exchanger, and a reboiler. Figure 1 shows the process of the absorption–desorption course of siloxanes using TPABr:TEG (1:3). The model polluted biogas stream containing a certain amount of methane and siloxanes is fed into the absorption column. The absorption process takes place under certain conditions maintained in the column (temperature of the process—Ta, the volume of DES—Va, flow rate of the biogas stream—wa). Pure methane from the top of the absorption column is collected. The next step in the entire process is desorbing the siloxanes from DES. For this purpose, the contaminated DES is directed into the stripper column which works in specific conditions (temperature of the stripper process—Ts, time of the stripper process—ts). Owing to regeneration, it is possible to reuse DES, which has a major impact on the economics of the process.

The absorptivity (*A*) of siloxanes in the TPABr:TEG (1:3) was calculated using Equation (2):

(2) $$A=\;\frac{C_{in}-C_{out}}{C_{in}}\;(\text{-})$$

where $c_{in}$—initial siloxanes concentration (mg/dm^3^), $c_{out}$—siloxanes concentration after absorption process (mg/dm^3^).

#### 2.3.5. Regeneration of DES

Following the selective absorption process of siloxanes, TPABr:TEG (1:3) was regenerated using nitrogen barbotage at an elevated temperature (80–100 °C). The regeneration process was carried out conducted in line with previous studies [24]. The regeneration experiments were conducted at 90 °C with an *N*_2_ flow of 50 mL/min. The concentration of L2, L3, and D4 (before and after regeneration) in TPABr:TEG (1:3) was studied by means of gas chromatography.

#### 2.3.6. Chromatographic Analysis

The degree and efficiency of the model biogas treatment were determined by gas chromatography coupled with a flame-ionization detector (GC-FID) (PerkinElmer, Waltham, MA, USA). The temperature of the GC oven was 120 °C, the detector temperature was 300 °C, the injection port temperature was 300 °C, the injection mode was split 5:1, and the carrier gas was nitrogen (2 mL/min).

#### 2.3.7. Physicochemical Properties of DES

##### FT-IR Analysis

FT-IR spectra were taken using attenuated total reflectance (ATR) with the following operating parameters: number of background scans: 256, number of sample scans: 256; spectral range: 4000–550 cm^−1^; resolution: 4 cm^−1^; and slit width: 0.5 cm.

##### Viscosity and Density Measurements

The viscosity and density of the synthesized TPABr:TEG (1:3) were measured within a temperature range of 25–60 °C. The uncertainty measurement for the temperature was 0.5 °C. Additionally, the relationship between the viscosity and revolutions per minute abbreviated (RPM) in the temperature range 25–60 °C was determined.

##### Melting Point Measurements

The melting point (MP) was determined visually at atmospheric pressure by cooling DES samples to −50 °C, followed by a temperature increase at 0.1 °C/min. The temperature at which the initiation of the phase transformation was observed was adopted as the melting point.

## 3. Results and Discussion

### 3.1. COSMO-RS Molecular Simulation

#### 3.1.1. Solubility of Siloxanes in DESs—Preselection of DES

**Figure 2:**
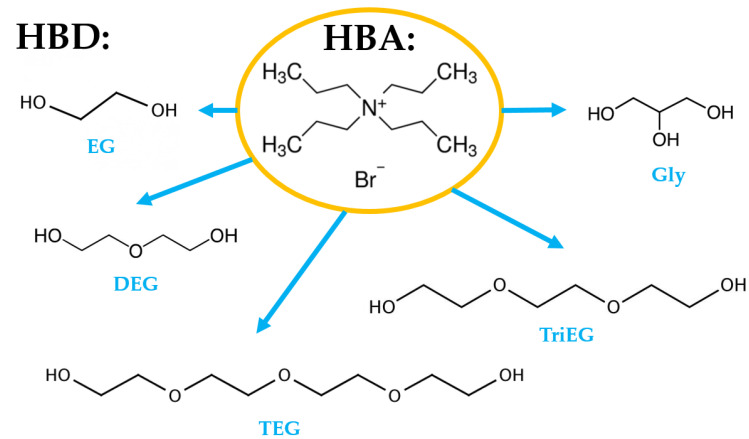
Structures of hydrogen bond acceptor (HBA) and hydrogen bond donors (HBDs).

The conductor-like screening model for real solvents (COSMO-RS) was used to calculate the solubility of siloxanes in pure glycols and water and in DESs composed with TPABr and glycols. Based on the previous studies, it can be deduced that COSMO-RS is a useful tool for solvent screening and predicting the impurities’ solubility in conventional as well as non-conventional solvents [35,41,42]. In most of the published works, the selection of solvents is made on certain parameters, i.e., Henry’s constant and activity coefficient. The results are often inconsistent. However, the most important parameter from the industrial point of view, solubility, is rarely reported [35,43]. Therefore, in this study, we calculated the solubility of individual siloxanes (L2, L3, and D4) in various DESs composed of TPABr as HBA and glycols, i.e., ethylene glycol (EG), glycerol (Gly), triethylene glycol (TriEG), tetraethylene glycol (TEG), and diethylene glycol (DEG), as HBD at various molar ratios (1:3; 1:4; 1:5; 1:6, HBA:HBD). These various molar ratios were selected on the basis of other studies which show that the melting point of most TPABr:glycols in 1:1, 1:2 complexes are higher than 20 °C [44,45]. This fact disqualifies the possibility of such DES as absorbents since one of the necessary conditions for absorbents is liquid at room temperature. The structures of HBA and HBDs are presented in Figure 2.

**Figure 3:**
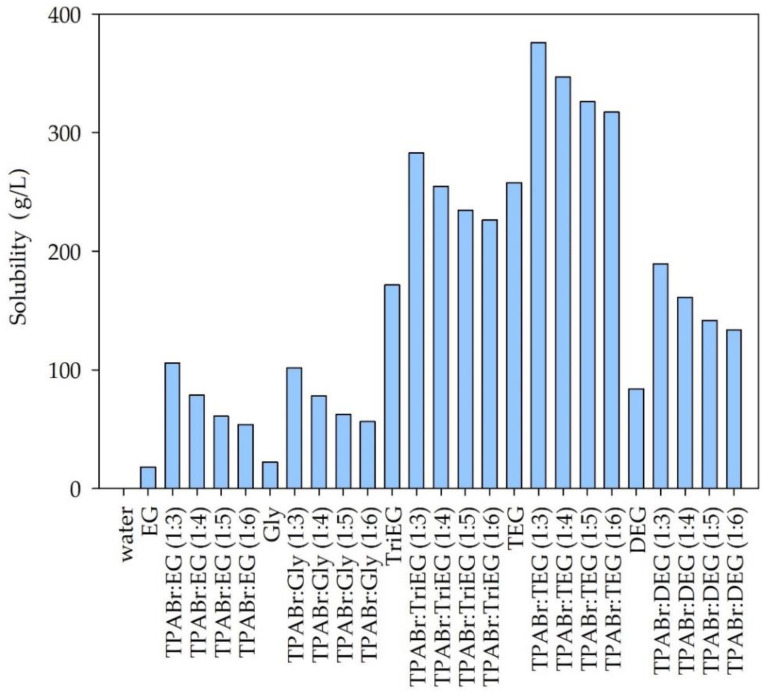
Solubility of hexamethyldisiloxane (L2) in DES and pure solvents.

**Figure 4:**
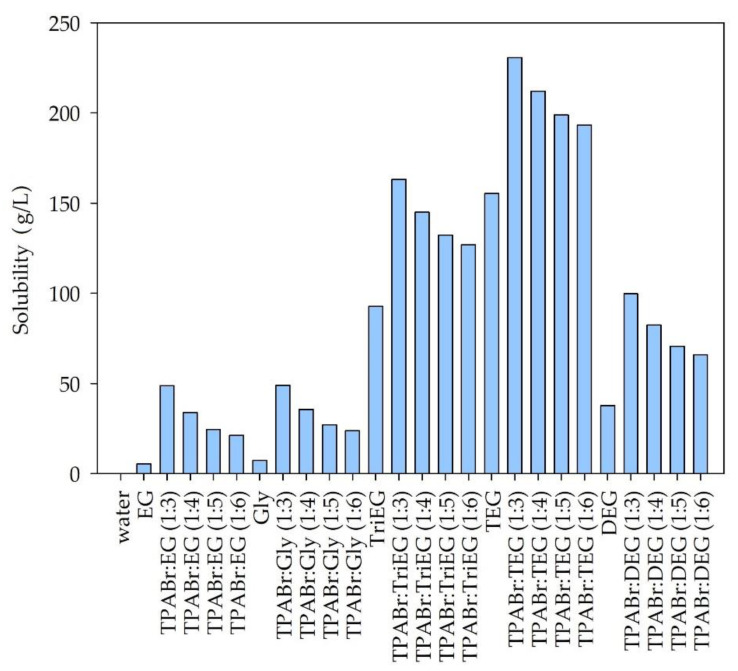
Solubility of octamethyltrisiloxane (L3) in DES and pure solvents.

**Figure 5:**
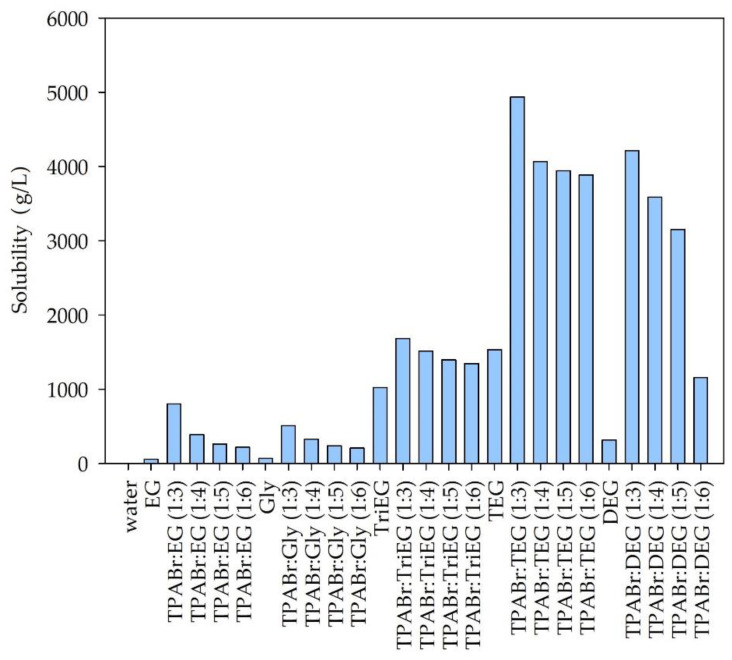
Solubility of octametylocyclotetrasiloxane (D4) in DES and pure solvents.

Additionally, the solubility of siloxanes in pure glycols and water were taken into account. The obtained results are presented in Figure 3, Figure 4 and Figure 5.

Among the tested solvents, water is the poorest solvent for siloxanes. The calculated solubility of individual siloxanes in water was 0.0054, 0.00011, and 0.027 g/L for L2, L3, and D4, respectively. This is due to the hydrophobic nature of most siloxanes [13,14]. From the industrial point of view, the ideal absorbents should be cheap and easily accessible. Due to the relatively high price of TPABr in comparison to the price of pure glycols, it would be advantageous to use pure EG, Gly, TriEG, TEG, or DEG to absorb siloxanes from biogas. However, the calculated solubility values are significantly lower for pure glycols compared to DES. The highest solubility can be observed for the DES composed of TPABr and glycols in 1:3 molar ratio. On the other hand, further increasing the amount of glycols in DES structures reduces the solubility of siloxanes. This indicates that both HBA and HBD take an active part in the absorption process by creating hydrogen or electrostatic bonds with siloxanes. COSMO-RS calculations indicate that D4, which represents cyclic siloxanes, shows higher solubility in DESs than linear siloxanes (L2 and L3). Similar results were obtained for ILs in the previous studies [46]. For linear siloxanes, as the length of the molecule decreases, their solubility in DESs increases. These are different results from those obtained for ionic liquids [46]. The highest solubility of both linear and cyclic siloxanes was obtained for DES composed of TPABr and TEG in 1:3 molar ratio. This is probably due to the formation of strong non-bonded interactions between TPABr:TEG (1:3) and siloxanes, i.e., hydrogen bonds between -OH group from TEG molecules, and O—a group from siloxanes. In order to obtain detailed information on the interactions between DES and siloxanes, analyses of σ-profiles and σ-potentials were performed. Due to the best siloxane dissolving ability of TPABr:TEG (1:3), only this DES was further investigated.

#### 3.1.2. σ-Profile

**Figure 6:**
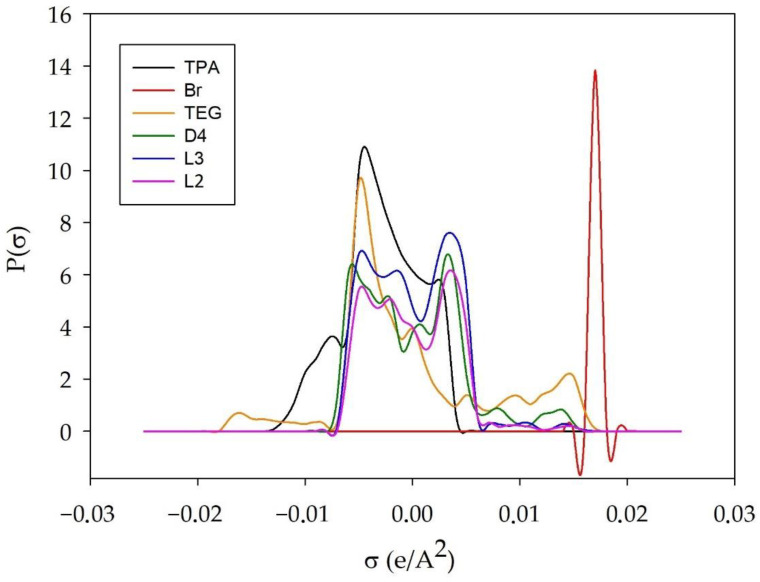
σ-Profile of TPABr:TEG (1:3), L2, L3, and L4.

A very important molecule-specific property in the COSMO-RS model is the σ-profile, which is the probability distribution of surface area with charge density (σ). Typically, σ-profile is presented as a histogram which can be divided into three regions i.e., HBA region σ > 0.0084 e/Å^2^; non-polar region −0.0084 e/Å^2^ < σ < 0.0084 e/Å^2^; and HBD region σ < −0.0084 e/Å^2^ [47]. The σ-profiles of TPA, Br, TEG, L2, L3, and L4 are shown in Figure 6.

The results indicate that the σ-profiles of all siloxanes are distributed within the non-polar and hydrogen bond acceptor region. There is no significant difference between σ-profiles of L2, L3, and D4. The only peak can be observed in the more positive region of the histogram for linear siloxanes. This indicates a slightly stronger hydrogen bond acceptor capacity of L2 and L3. Similar results were observed in other studies [35,46]. The distribution of TPA shows the concentration of the charge density mainly around 0, and a small concentration of the charge below −0.0084, which indicates the role of TPA as a hydrogen bond acceptor in hydrogen bond formation. The distribution of the bromide anion is located around 0.018 in the HBA area, which demonstrates a non-polar character and the possibility of H-bonding formation. On the other hand, the distribution of TEG is observed over the entire range of the σ-profile. This indicates that TEG can be both an acceptor and a hydrogen bond donor.

#### 3.1.3. σ-Potential

**Figure 7:**
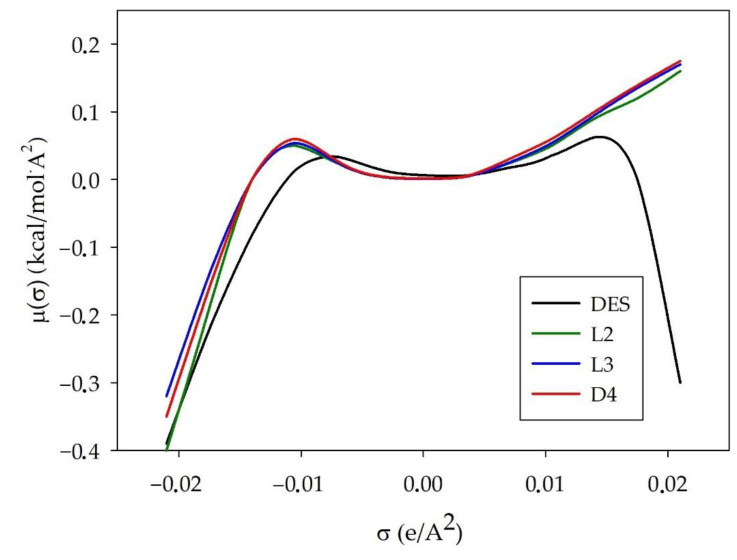
σ-Potential of TPAB:TEG (1:3) L2, L3, and L4.

The σ-potential is typically used to indicate the affinity between mixture components. The higher values of the positive μ (σ) suggest an increase in its repulsive behavior, and higher negative values of the μ (σ) indicate a stronger interaction between the molecules. Similarly to the σ-profile plot, the σ-potential plot is divided at the same three regions. The σ-potential for TPABr:TEG (1:3), L2, L3, and D4 are plotted in Figure 7. The obtained results indicate that all siloxanes almost overlap each other, which means that L2, L3, and D4 have similar molecular interaction properties with other molecules and with themselves. The shape of siloxanes σ-potential is negative in the HBA region and positive in the HBD region. This means that L2, L3, and D4 can be acceptors in H-bonding formation. However, the DES shape is negative in both these regions. This indicates that it is both an acceptor and a hydrogen bond donor. Therefore, the formation of hydrogen bonds is the most likely driving force in the process of removing siloxanes from biogas.

### 3.2. Structural and Physicochemical Properties of DES

#### 3.2.1. FT-IR Analysis

**Figure 8:**
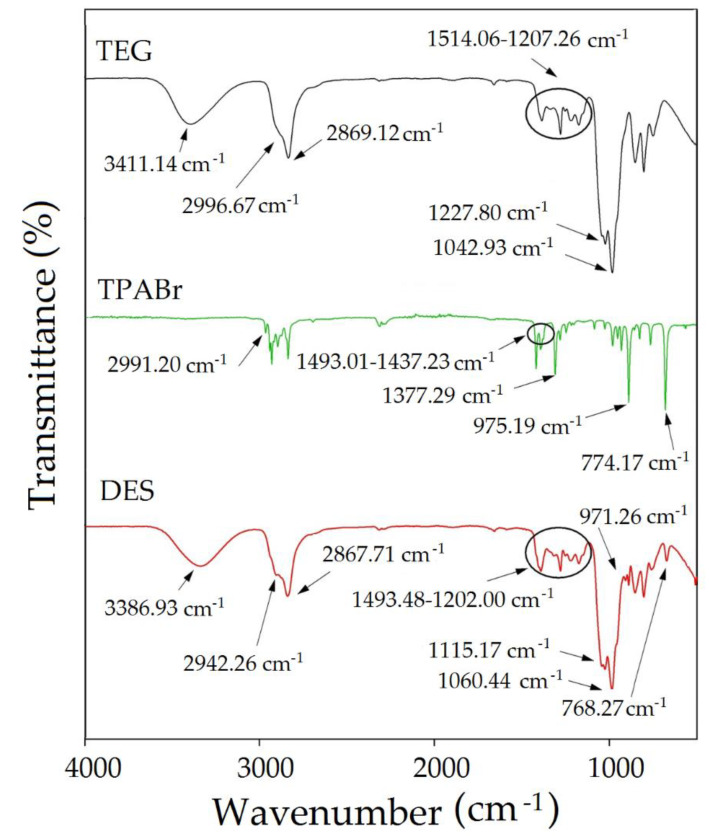
FT-IR spectrum for pure TPABr, TEG, and TPABr:TEG (1:3).

Spectroscopic characterization is a very important aspect to determine the interaction between HBA (TPABr) and HBD (TEG). For this purpose, the FT-IR analysis was used in the study (Figure 8).

Figure 8 shows the mechanism of TBABr:TEG (1:3) formation. In the TPABr:TEG (1:3) spectrum, the shift of -OH group vibration towards lower values compared with pure TEG HBD (from 3411.14 to 3386.93 cm^−1^) indicates the formation of O-H O or O-H Cl bonds. In addition, the broadening and shifts of the vibration towards lower values of the aliphatic C–H stretching bonds (from 2996.67 and 2869.12 cm^−1^ to 2942.26 and 2867.71 cm^−1^) can be observed. The shifts O-H O or O-H Cl, and C-H groups are most likely the consequence of hydrogen bond formation between TPABr and TEG [48,49]. Moreover, shifts of the OH group may result from the presence of C-O-C groups in the TEG. The C-O-C group are considered as the electronegative groups and tend to attract electrons on hydrogen in OH bands. The occurring interactions between TPABr and TEG can be confirmed by the shift of the C-O-C group towards lower values from 1227.80 to 1115.17 cm^−1^ and increasing the intensity of the OH group [50]. Similar vibration towards lower values can be seen in the peaks in the bands responding with H-bending, CH_2_ deformation, and N-C-C bending bonds from 1514.06–1207.26 cm^−1^ to 1493.48–1202.00 cm^−1^, and C-N bond symmetric stretching vibration from 774.17 to 768.27 cm^−1^ as well as redshift phenomena O-H and C-O-H stretching bonds from 1042.73 to 1060.44 cm^−1^. The shifts confirm the interaction between the atoms in TPABr and TEG [51,52,53].

#### 3.2.2. Viscosity and Density Measurements

**Figure 9:**
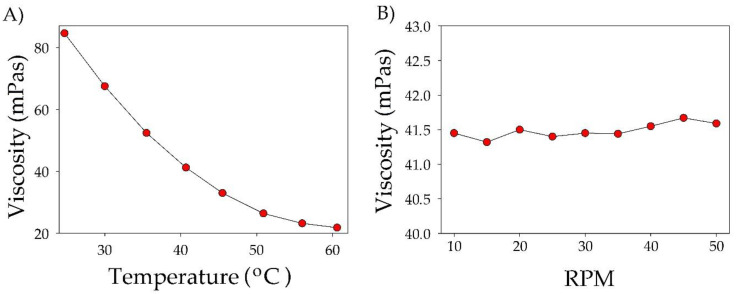
(**A**) Viscosity for TPABr:TEG (1:3) as a function of temperature in the range 25–60 °C. (**B**) Dependence of viscosity on turnover in the range 10–50 RPM in temperature 40 °C.

It is well known that DES components and temperature have a dramatic effect on the absorbent density and viscosity. Basic physicochemical parameters of DES strongly influence the ability of the mass transfer capacity, which is of great importance for any changes in the absorption process [54,55]. In order to analyze the flow behavior of synthesized TPABr:TEG (1:3), the viscosity was studied in a function of shear rate ranging 10–50 rpm and temperature range 25–60 °C. The obtained results indicate that the viscosity of TBABr:TEG (1:3) decreases with increasing temperature. The increase in temperature causes the velocity of the particles in the liquid to increase, which reduces the intermolecular forces, resulting in a decrease in the TPABr:TEG (1:3) viscosity (Figure 9A). At room temperature, the viscosity of TPABr:TEG (1:3) is 84.6 mPas; it should be noted that it is much lower compared to the DESs which are presented in the literature. The dynamic viscosity of DES composed of tetrabutylammonium bromide (TBABr) and glycerol (Gly) or ethylene glycol (EG) in a molar ratio of 1:3 were 467.2 and 91.4 mPas, respectively [56]. A decrease in the viscosity value contributes to the increase in the capacity and rate of absorption because it makes the mass transfer easier. Therefore, DESs with lower value viscosities are more desirable for absorption processes.

In Figure 9B, it can be observed that the viscosity of TPABr:TEG (1:3) remains almost constant throughout the range of the applied shear rate ranging. Therefore, it can be concluded that the obtained DES is a Newtonian liquid [57]. The possible shear thinning behavior can be attributed to different strengths of the H-bonding present in TPABr:TEG (1:3) which can start breaking with increasing RPM. However, deeper analysis is required to confirm these assumptions. Similar behavior was also observed for another type of DES [58].

**Figure 10:**
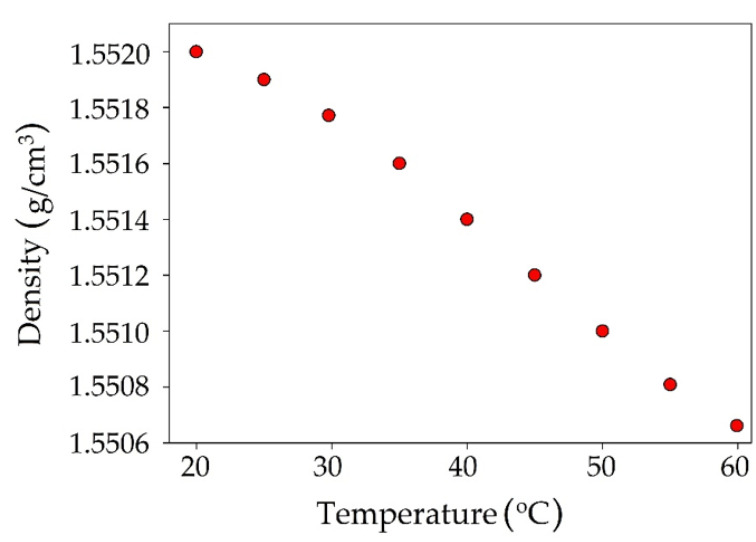
Density for TPABr:TEG (1:3) as a function of temperature in the range 25–60 °C.

Another tested physicochemical parameter was density. The value of DES density decreases linearly with increasing temperature (Figure 10). At 20 °C, the density TPABr:TEG (1:3) is 1.5520 g/cm^3^. However, it can be observed that with increased temperature (60 °C), the density value decreases to 1.5508 g/cm^3^. The lower density values can be due to the fact that during heating, HBA and HBD in DES vibrate harder. These vibrates can cause molecular rearrangements between HBA and HBD, which can contribute to creating weaker interactions in the hydrogen bonding [59]. The obtained density of TPABr:TEG (1:3) is higher compared to the DESs which are composed of quaternary ammonium salts (ChCl or TBABr) [56,60].

#### 3.2.3. Melting Point Measurements

The measured MP of TPABr:TEG (1:3) is −48 °C. As expected, the melting point of TPABr:TEG (1:3) is lower than the MP of pure TEG (−9.4 °C) [61]. The depression in the melting point of the mixture shows the formation of strong intermolecular interactions, i.e., hydrogen bonds between TPABr and TEG [62].

### 3.3. An Experimental Studies on Absorption of Siloxane Compounds

#### Optimization of the Absorption Process Conditions

In our research, the processes of absorption using a new DES based on TPABr:TEG (1:3) were carried out for purification of the model biogas stream from L2, L3, and D4 pollutants. The absorption processes were optimized in terms of the volume of TPABr:TEG (1:3), model biogas flow, and temperature.

**Figure 11:**
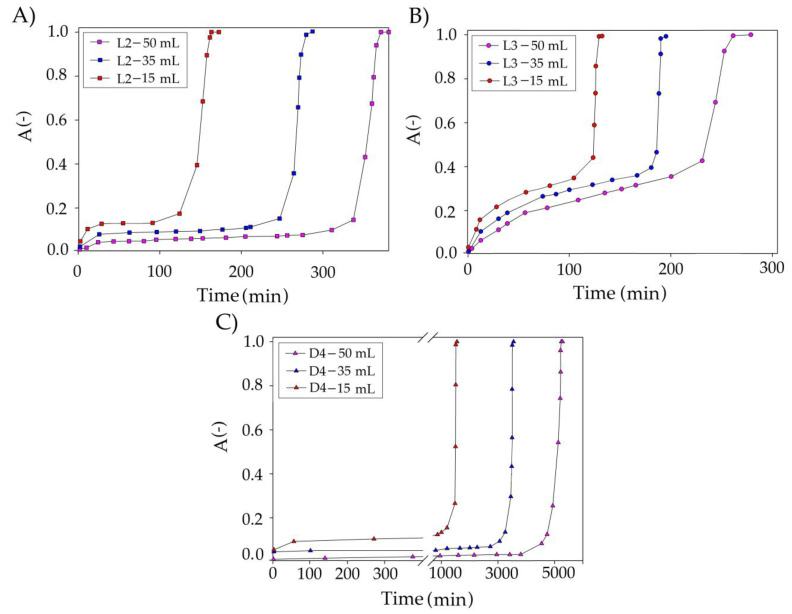
Experimental breakthrough curves of siloxane absorption with TPABr:TEG (1:3) at the different volumes of DES for (**A**) L2; (**B**) L3; and (**C**) D4.

The first optimized parameter was the volume of the TPABr:TEG (1:3) in the range of 15–50 mL/min (Figure 11). The results show that the volume of DES has a significant impact on the overall siloxane capture process. As the volume of DES increases from 15 to 50 mL/min, the DES saturation time increased from 150 to 320 min (L3—Figure 11B), from 140 to 400 min (L2—Figure 11A), and from 1551 to 5281 min (D4—Figure 11C). The increase in saturation time can be explained by increases in the contact time between the siloxane gas phase and the absorbent [63]. Increasing the volume of DES also contributes to an increase in the amount of active substance (TPABr:TEG (1:3)) and an increase in the number of active sites that are responsible for capturing of the siloxanes from DES.

**Figure 12:**
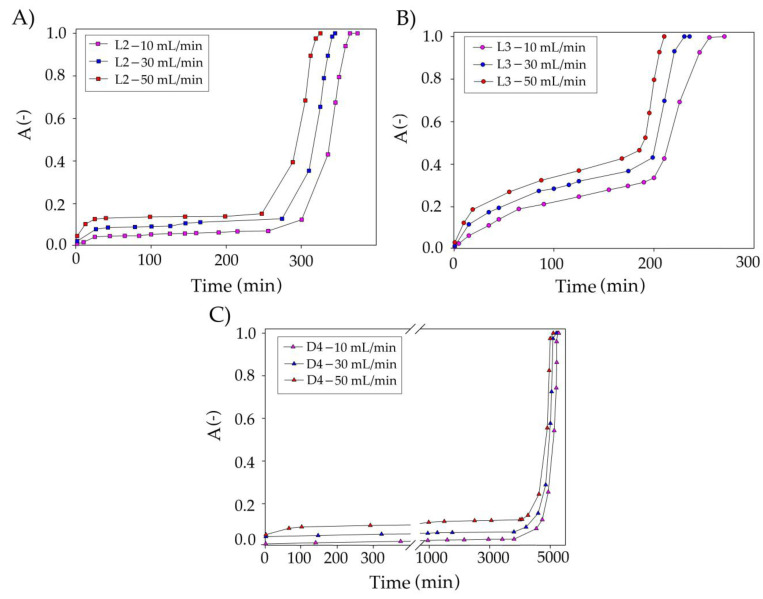
Experimental breakthrough curves of siloxane absorption with TPABr:TEG (1:3) at the different biogas flow rate for (**A**) L2; (**B**) L3; and (**C**) D4.

The next studied parameter was model biogas flow rate in the range of 10–50 mL/min (Figure 12). The results indicate that the flow rate has only a minor effect on siloxane uptake compared to DES volume. The conducted research indicates that an increase in the flow rate from 10 to 50 mL/min slightly decreased the effectiveness of siloxane removal from the model biogas stream. Similar results were observed in the previous studies [29,64]. In the industrial technologies used with the use of a water scrubber, a flow of 88 mL/min is used to remove CO_2_ or H_2_S [65], whereas when using an amine scrubber, flows of 30 mL/min are used [66]. The reduction in the flow rate may result from the different viscosities of the use of the absorbent. Therefore, the assumed optimal value of 50 mL/min seems to be the rational and comparable value.

**Figure 13:**
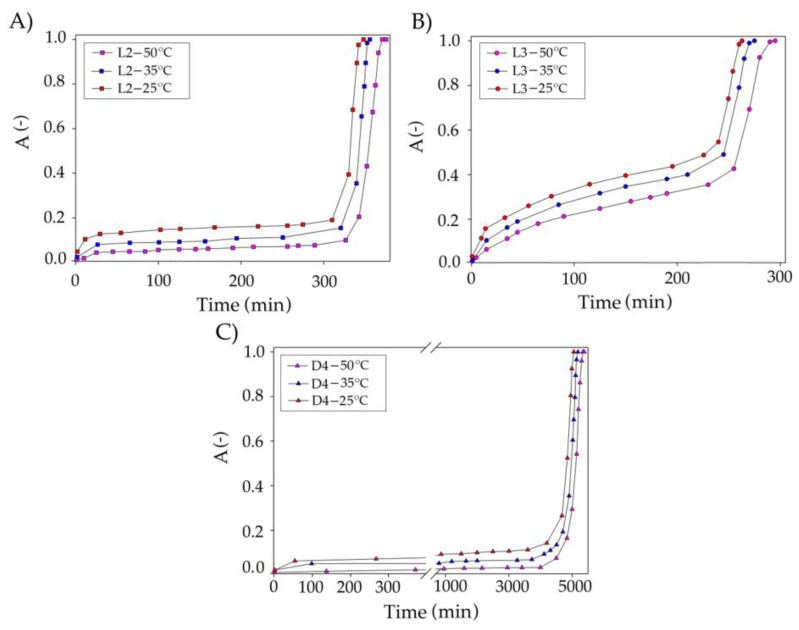
Experimental breakthrough curves of siloxane absorption with TPABr:TEG (1:3) at different temperatures for (**A**) L2; (**B**) L3; and (**C**) D4.

The temperature in the range of 25–50 °C was selected as the last parameter for optimizing the absorption conditions (Figure 13). An increase in temperature causes decreases in TPABr:TEG (1:3) viscosity. The lower viscosity improves the mass transfer efficiency and, hence, the siloxane removal efficiency should be higher. However, increasing the temperature does not extend the absorption process too much. This is likely due to the fact that the absorption process is normally exothermic [67]. Therefore, a temperature of 25 °C was adopted as optimal.

**Figure 14:**
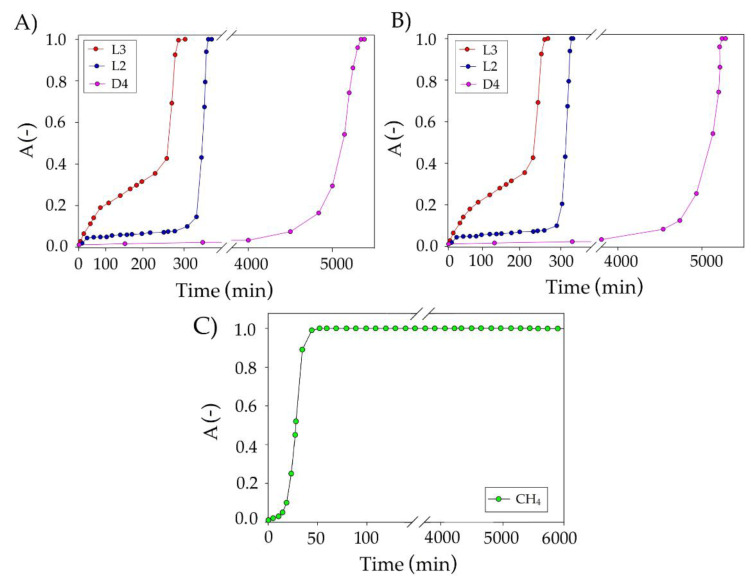
Experimental breakthrough curves of impurities absorption with TPABr:TEG (1:3) using (**A**) pure nitrogen and (**B**) mixture of nitrogen and methane at 2:1 volume ratio under optimum conditions, and (**C**) methane absorption curve using TPABr:TEG (1:3).

Owing to the conducted research, the optimum conditions for the removal of siloxanes from the model biogas stream were selected as a temperature of 25 °C, a DES volume of 50 mL, and a flow rate of 50 mL/min. The obtained dependence of the absorption efficiency on the duration of the absorption process of individual pollutants is shown in Figure 14A (with the use of pure nitrogen) and Figure 14B (with the use of a mixture of nitrogen and methane gases in the volume ratio 2:1).

For D4 in pure *N*_2_, after 5380 min of absorption process, a sharp increase in the supersaturation value was observed. While for D4 in the mixture of nitrogen:methane at 2:1 volume ratio, the saturation time was 5300 min. The oversaturation times of the other two siloxanes in *N*_2_ were 400 and 300 min, while in *N*_2_:CH_4_ (2:1), they were 375 and 280 min, respectively, for L2 and L3. In the literature, there are very few works that focus on the capture of siloxanes from biogas. The results obtained in our studies can only be compared to the absorption in which the absorbent consists of amines, acids, or bases. However, it is well known that the strong bases and acids contribute to the cleavage of Si-O bonds and convert siloxanes to other volatile compounds with lower boiling points [68].

Devia and Subrenat [15] proposed L2 and D4 absorption into motor oil, cutting oil, and water-cutting oil. The studies showed the best results were obtained for motor oil for which the breakthrough curves obtained to allow for efficient removal of siloxanes were for 191.4 min (L2) and for 47.1 min (D4). The obtained results show that the proposed new DES-based absorbents show a much higher absorption capacity towards siloxanes than conventional solvents. In the studies, apart from monitoring the siloxane absorption process, the concentration of methane was also monitored (Figure 14C). The results show that complete saturation of TPABr:TEG (1:3) with methane occurs after 50 min of the process. The loss of methane in the entire process of siloxane absorption was within 5%.

### 3.4. FT-IR Studies on Absorption of Siloxane Compounds

**Figure 15:**
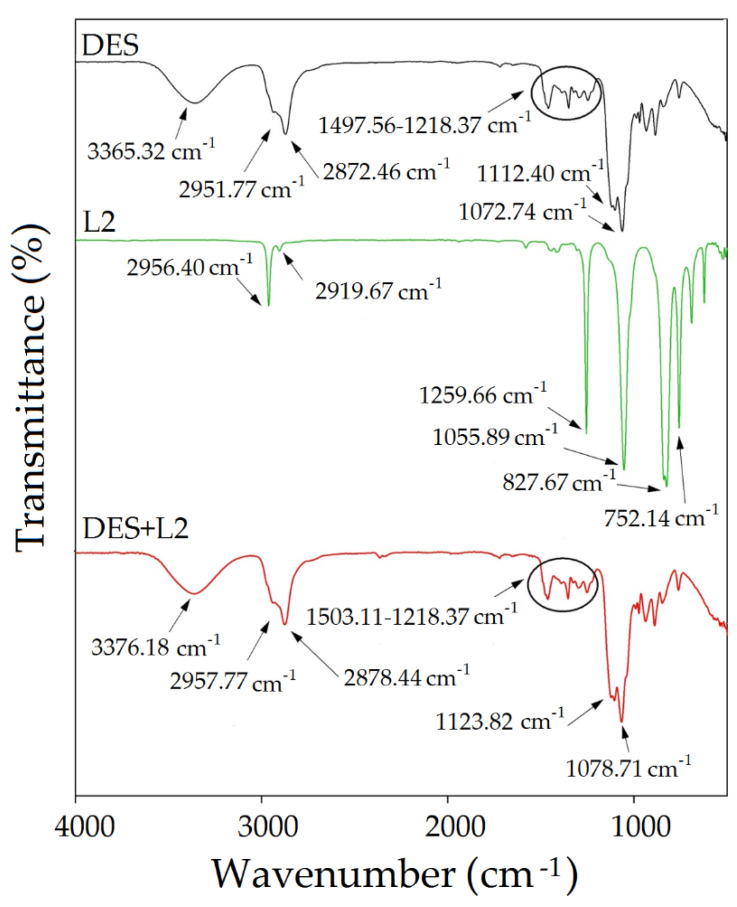
FT-IR spectrum for pure TPABr:TEG (1:3), pure L2 and complex TPABr:TEG + L2 (DES + L2).

**Figure 16:**
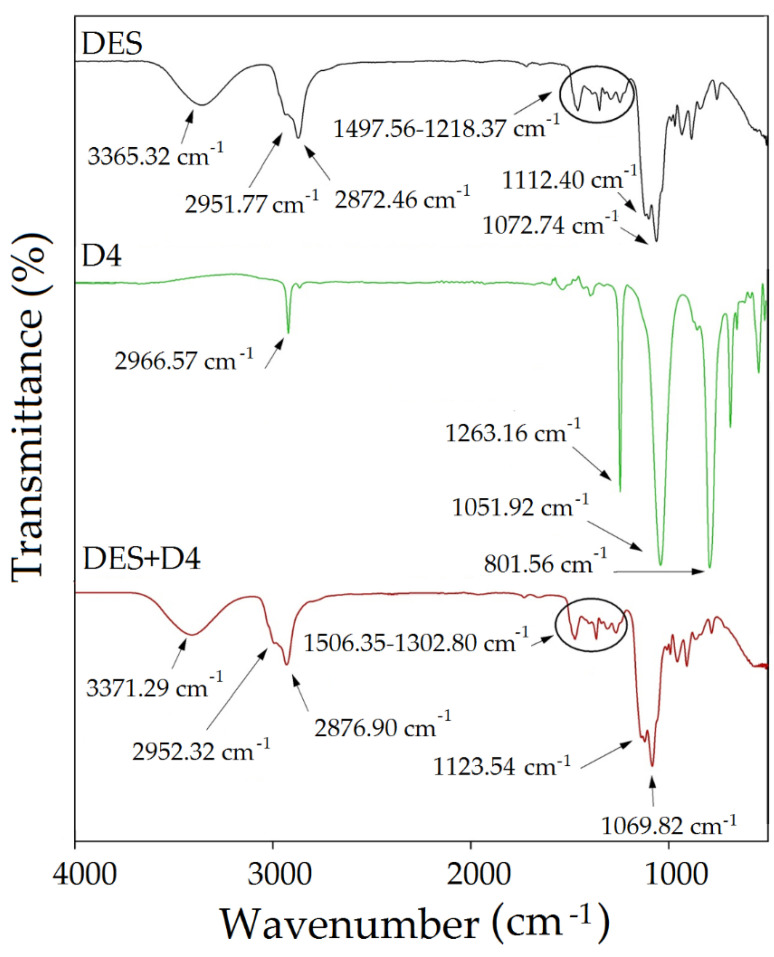
FT-IR spectrum for pure TPABr:TEG (1:3), pure D4 and complex TPABr:TEG + D4 (DES + D4).

**Figure 17:**
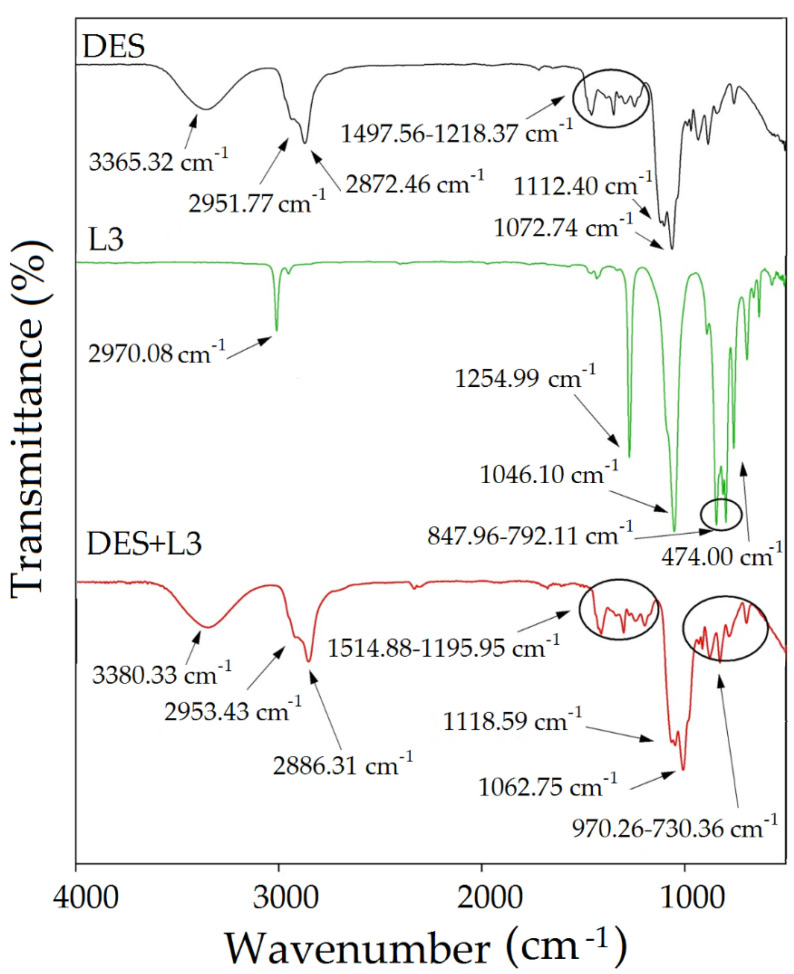
FT-IR spectrum for pure TPABr:TEG (1:3), pure L3 and complex TPABr:TEG + L3 (DES + L3).

The experimental study on the mechanism of the absorption process of siloxanes was conducted by FT-IR analysis. The obtained spectra of pure TPABr:TEG (1:3) and pure siloxanes were compared with the spectra of TPABr:TEG (1:3) after the absorption process (Figure 15, Figure 16 and Figure 17). All of them identified the bands which can be observed in the FT-IR spectrum for pure siloxanes: Si-O-Si antisymmetric stretch bonds in the range 1000–1100 cm^−1^ and Si-C symmetric stretch bonds at 800 cm^−1^ are visible in the spectrum of the TPABr:TEG (1:3) after the absorption process [69]. In the spectrum after the absorption process, new peaks or significant band shifts cannot be observed. Only shifts of the -OH stretching vibration and aliphatic C-H stretching bonds are visible, which confirms the phenomena of physical absorptions. In addition, the shifts of -OH stretching vibration indicate that the hydroxyl group from TPABr:TEG (1:3) may interact with the oxygen atoms from siloxanes by forming hydrogen bonds, which is in accordance with the siloxane absorption [70]. Additionally, a shift of the bands originating from group C-O-C towards higher values from 1112.40 to 1123.83 cm^−1^ (Figure 15), 1123.54 cm^−1^ (Figure 16), and 1118.59 cm^−1^ (Figure 17) are observed. These shifts indicate that siloxane absorption can also occur through the interaction of silicon atom (Si-OH—827.67 cm^−1^ and SiO—752.14 cm^−1^ (Figure 15), Si-O—801.56 cm^−1^ (Figure 16), Si-OH and Si-O in the range 847.96–792.11 cm^−1^ (Figure 17)) with the oxygen atoms with C-O-C in the DES (1:3) [50].

### 3.5. Regeneration of DES

**Figure 18:**
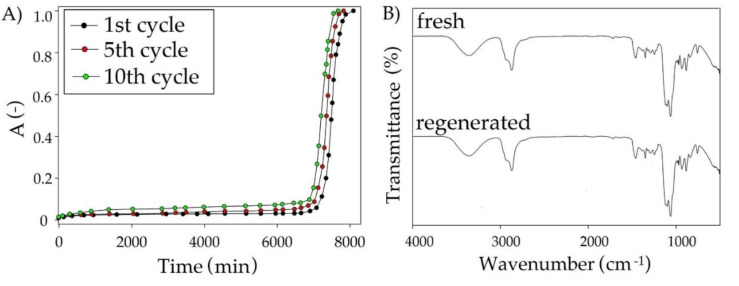
(**A**) Reusability of TPABr:TEG (1:3) on the example of removing L2, (**B**) FT-IR spectra from on fresh and regenerated TPABr:TEG (1:3).

From the industrial point of view, the regeneration processes of absorbents are extremely important because they determine the final costs. The obtained results indicate that siloxanes can be completely removed from TPABr:TEG (1:3) using nitrogen barbotage conducted at a temperature of 90 °C for 3 h. TPABr:TEG (1:3) shows tall and almost unchanging L2, L3, and D4 removal efficiency for up to 10 regeneration cycles (Figure 18A). In addition, the thermal stability of TPABr:TEG (1:3) by means of FT-IR analysis was confirmed. The comparison of fresh and regenerated TPABr:TEG (1:3) spectrum indicates a lack of additional shifts and peaks in the regenerated TPABr:TEG (1:3) (Figure 18B). This confirms stability and effective regeneration of TPABr:TEG (1:3).

## 4. Conclusions

In the paper, the solubility of siloxanes (L2, L3, and D4) in deep eutectic solvents (DESs) composed of tetrabutylammonium bromide and glycols as well as conventional solvents was investigated. The siloxane solubility was predicted by means of the COSMO-RS model where the highest solubility of both linear and cyclic siloxanes was present in the DES composed of TPABr and TEG in a 1:3 molar ratio. The chemical structures of TPABr:TEG (1:3) and the interaction structures between TPABr and TEG as well as between DES and siloxanes were reported using FT-IR spectroscopy. Furthermore, in order to confirm the interactions, the analyses of σ-profiles and σ-potentials were used.

The results of physicochemical properties indicate that TPABr:TEG (1:3) is a Newtonian liquid due to the lack of viscosity changes during shear changes, which contribute to only minor changes in siloxane removal efficiency with increasing temperature. In turn, carrying out the absorption process at a temperature of 25 °C is beneficial from an economic point of view. Under optimum conditions (50 mL of TPABr:TEG (1:3), 50 mL/min flow rate, and temperature 25 °C), the L2, L3, and D4 can be removed with high efficiency for 375, 280, and 5300 min, respectively. These are much better absorption efficiencies compared to mineral oils. In addition, TPABr:TEG (1:3) also can be easily regenerated up to 5 cycles without significantly changing the siloxane absorption efficiency. The studies on the absorptive mechanism to remove siloxanes indicate that the reason for the high solubility siloxanes in TPABr:TEG (1:3) is the formation of the strong hydrogen interactions between -OH group from DES molecule, and -O- a group from siloxanes.

The cost of the absorption process mainly depends on the type of absorbents. The estimated capital cost of the absorption process based on TPABr:TEG (1:3) is 126.05 €/L [71,72]. The DES price is higher than conventional absorbents prices which are 9.17 €/L for motor oil 5W40 from Elf) [73]; 33.42 €/L for cutting oil Hochleistungs-Schneidöl Alpha 93 from Jokish^®^ GmbH [74]. However, it should be remembered that DES can be used for up to 5 cycles without changing the high efficiency of removing siloxanes from biogas. The initial price of 126.05 €/L can drop to 25.21 €/L. Therefore, DESs can be used as alternative absorbents.

## Data Availability

The data presented in this study are available on request from the corresponding author.
